# Supplementary material for: Distant from input: Evidence of regions within the default mode network supporting perceptually-decoupled and conceptually-guided cognition
Source: Neuroimage. 2018 May 1;171:393–401. doi: 10.1016/j.neuroimage.2018.01.017 (PMC5883322; doi:10.1016/j.neuroimage.2018.01.017)
Supplement: NeuroImage_Manuscript_Revisions_Jan9th.docx [33] [file mmc1.docx]

***Supplementary material***

**Supplementary Table 1. Behavioural Results.**

| Condition | RT | | Accuracy | |
| --- | --- | --- | --- | --- |
|  | Mean (ms) | SE | Mean  (% correct) | SE |
| Colour 1-back | 998.59 | 44.86 | 97.15 | 2.72 |
| Colour 0-back | 796.34 | 47.62 | 96.04 | 2.90 |
| Object 1-back | 1003.64 | 48.34 | 96.42 | 2.92 |
| Object 0-back | 802.18 | 47.98 | 95.38 | 2.87 |

Footnote: SE = standard error, ms = milliseconds, % = percentage.

Supplementary Table 2. Univariate Results.

| Contrast | Region | Voxels | Z | x | y | z |
| --- | --- | --- | --- | --- | --- | --- |
| 0-> 1-back | R. Temporal occipital fusiform cortex | 943 | 4.78 | 28 | -50 | -16 |
|  | L. Temporal occipital fusiform cortex | 870 | 5.74 | -28 | -50 | -18 |
|  | L. Lateral occipital cortex, inferior | 150 | 4.56 | -40 | -84 | -2 |
| 1 > 0-back | L. Supracalcarine cortex/ precuneous | 1452 | 5.04 | -12 | -68 | 18 |
|  | L. supramarginal gyrus, posterior /angular gyrus | 870 | 4.17 | -62 | -50 | 18 |
|  | L. Middle temporal gyrus, posterior | 637 | 4.88 | -60 | -22 | -12 |
|  | R. Parietal operculum cortex | 174 | 4.10 | 46 | -28 | 20 |
|  | R. Lateral occipital cortex | 143 | 3.79 | 42 | -78 | 36 |
| Object>Colour | R. Superior frontal gyrus | 1010 | 6.38 | 6 | 54 | 26 |
|  | R. Lateral occipital cortex, superior | 608 | 4.35 | 52 | -64 | 26 |
|  | R. Putamen | 601 | 4.32 | 18 | 4 | -8 |
|  | R. Middle frontal gyrus | 266 | 3.89 | 38 | 16 | 44 |
|  | L. Lateral occipital cortex, superior | 197 | 3.86 | -42 | -66 | 36 |
|  | L. Middle temporal gyrus, posterior | 159 | 4.17 | -62 | -26 | -18 |
| Object>Colour |  |  |  |  |  |  |

Footnote: R = right hemisphere, L = left hemisphere. Cluster corrected at Z = 3.1 FWE. No significant clusters for object > colour conditions.

**Supplementary Figure 1..** *Comparison of complex memory representation in the presence or absence of relevant perceptual input.* Spatial maps were cluster corrected at Z = 3.1 FWE.
